# Current Practice in Using Voltage Imaging to Record Fast Neuronal Activity: Successful Examples from Invertebrate to Mammalian Studies

**DOI:** 10.3390/bios13060648

**Published:** 2023-06-13

**Authors:** Nikolay Aseyev, Violetta Ivanova, Pavel Balaban, Evgeny Nikitin

**Affiliations:** Institute of Higher Nervous Activity and Neurophysiology, Russian Academy of Sciences, Butlerova 5A, Moscow 117485, Russia; aseyev@ihna.ru (N.A.); v.o.ivanova@ihna.ru (V.I.); pmbalaban@gmail.com (P.B.)

**Keywords:** voltage-sensitive dyes, voltage imaging, GEVI, VSD, neuron, neuronal activity, optical recording

## Abstract

The optical imaging of neuronal activity with potentiometric probes has been credited with being able to address key questions in neuroscience via the simultaneous recording of many neurons. This technique, which was pioneered 50 years ago, has allowed researchers to study the dynamics of neural activity, from tiny subthreshold synaptic events in the axon and dendrites at the subcellular level to the fluctuation of field potentials and how they spread across large areas of the brain. Initially, synthetic voltage-sensitive dyes (VSDs) were applied directly to brain tissue via staining, but recent advances in transgenic methods now allow the expression of genetically encoded voltage indicators (GEVIs), specifically in selected neuron types. However, voltage imaging is technically difficult and limited by several methodological constraints that determine its applicability in a given type of experiment. The prevalence of this method is far from being comparable to patch clamp voltage recording or similar routine methods in neuroscience research. There are more than twice as many studies on VSDs as there are on GEVIs. As can be seen from the majority of the papers, most of them are either methodological ones or reviews. However, potentiometric imaging is able to address key questions in neuroscience by recording most or many neurons simultaneously, thus providing unique information that cannot be obtained via other methods. Different types of optical voltage indicators have their advantages and limitations, which we focus on in detail. Here, we summarize the experience of the scientific community in the application of voltage imaging and try to evaluate the contribution of this method to neuroscience research.

## 1. Introduction

### 1.1. Outline 

The ability to simultaneously record fast electrical activity from many neurons or local areas of the brain has been a dream of neuroscientists for decades. The first suitable voltage sensitive dye, merocyanine 540, was developed in 1972 by Larry Cohen and Brian Salzberg [1]. Using epifluorescent imaging with a VSD, the pioneering work from Cohen’s lab [2] ushered in the era of optical recording of fast neuronal activity and inspired many groups of scientists to employ this method in their neuroscience research. Our group joined the field to apply voltage imaging in our studies of plasticity and memory. Here, we summarize the experience of the scientific community with the application of voltage imaging and attempt to assess the contribution of the method to neuroscience research.

### 1.2. Comparison of VSD vs. GEVIs

Membrane potential recording with electrodes that most neurophysiologists rely on has many limitations. The electrical properties of dendritic spines, synapses, and axonal terminals are difficult to measure because they are small. Voltage imaging, because it is relatively non-invasive and could work at low and high optical magnifications, seems to be an ideal solution for recording membrane potentials in both spines and circuits [3]. The progress in the development of VSD leads to the use of different classes of substances, such as ANNEPS dyes [4], ANNINE dyes [5], ICG dye [6], and the PeT family of fluorescent sensors [7,8]. NewANNINE-6 dyes are suitable for the two-photon imaging of single cells in vivo [9]. New promising dyes have recently been synthesized [10,11]. Current advances in VSD design include near-infrared sensors that are compatible with optogenetic tools, such as channelrhodopsin optical stimulation [12], new methods for targeting neurons of interest [13,14], and the development of the new dyes for photoacoustic recording [15,16].

**Figure 1:**
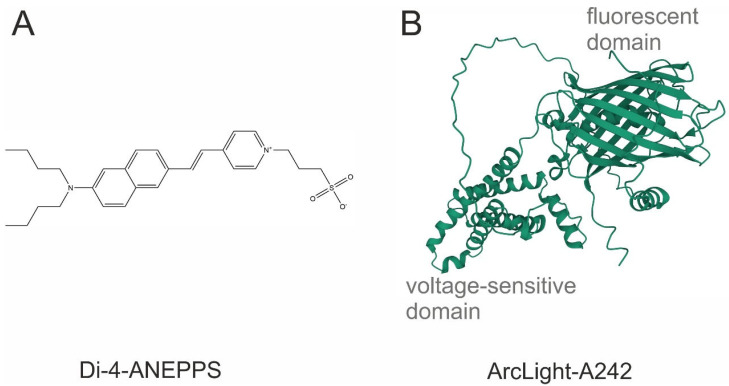
The chemical structure of the most common potentiometric sensors. (**A**) Di-4-ANEPPS, a VSD; (**B**) ArcLight A242, a GEVI. Sequence obtained from [20]. Three-dimensional view was generated with AlphaFold2.

The genetically encoded voltage indicator (GEVI) is an artificial membrane protein that is genetically targeted to the cells of interest and can change their level of fluorescence in response to changes in membrane potential (Figure 1). The first GEVI introduced was a synthetic protein made during the fusion of a voltage-gated channel and a fluorescent protein [17], and it was too slow to achieve single-action potential (AP) temporal resolution. The second generation of GEVIs, based on the fusion of *Ciona* voltage-sensitive phosphatase as a potential sensitive domain, provides better targeting to the plasma membrane and may have fluorescent domains operating at different wavelengths [18,19]. Subsequent advances in GEVI design have led to an improved temporal resolution and an increased magnitude of responses, enabling single AP detection in the third generation of GEVIs [20,21]. Recently, the targeted mutation tuning of the ArcLight-based sensor has improved the temporal parameters and magnitude of the GEVI [22], making it almost comparable to VSDs in terms of signal magnitude, but still inferior to VSDs in terms of speed.

There are also hybrid voltage sensors (hVOS) that work via the FRET interaction mechanism between two genetically encoded fluorescent proteins (or a fluorescent protein and a small lipophilic molecule), which also gives the possibility to record APs in a single trial [23,24,25,26,27]. Reversing the action of bacterial rhodopsins allows them to sense the voltage via fluorescence changes, which is the principle of opsin-based GEVIs [28,29]. This class of GEVIs is relatively dim and requires much higher excitation light intensities, but exhibits unprecedentedly fast response dynamics. To date, new GEVIs have been empirically designed via laborious mutagenesis and screening [30,31,32,33], and the theory behind the process seems to be emerging [34].

The main advantage of small molecule VSDs for recording fast neuronal activity is a good signal-to-noise ratio and excellent temporal resolution, allowing the study of postsynaptic potentials and AP propagation through neurites and neuronal networks in ex vivo preparations. It is also possible to record post-synaptic potentials and APs in vivo using VSD ANNINE-6plus and two-photon microscopy [35,36]. A relatively simple bath application or the more difficult patch pipette loading technique does not require established viral transfection techniques in the laboratory or the use of transgenic animals. VSD recording may be the method of choice for potential imaging in genetically intractable animal models, such as many invertebrates or lower vertebrates. Electrochromic voltage-sensitive dyes show a spectral shift for both excitation and emission, while pure electrochromic dyes (ANNINE-6 and ANNINE-6plus) show the same spectral shifts for both excitation and emission on an energy proportional scale [3,9]. A disadvantage of using VSD, which works via an electrochromism mechanism, is the necessity to carefully choose excitation and emission filters for the specific dye, as the signal arises from only a minor spectral shift. In the case of GEVIs based on GFP or other well-characterized fluorescent proteins, a convenient set of filters can be used for acquisition.

The main advantage of GEVIs is the possibility to make the protein sensor target the plasma membrane of specific cell populations [37,38,39] or cell compartments [40] via choosing the promoter in ex vivo and in vivo experiments in genetically tractable animal models. Recent advances in GEVI design allow researchers to perform fast neuronal recording (with single AP resolution), although temporal parameters are still inferior compared to VSDs, but they are superior to calcium sensors [41]. Two recent comprehensive studies focused on the comparison of different available sensors and are recommended for the rapid selection of GEVIs for a particular model [41,42].

**Figure 2:**
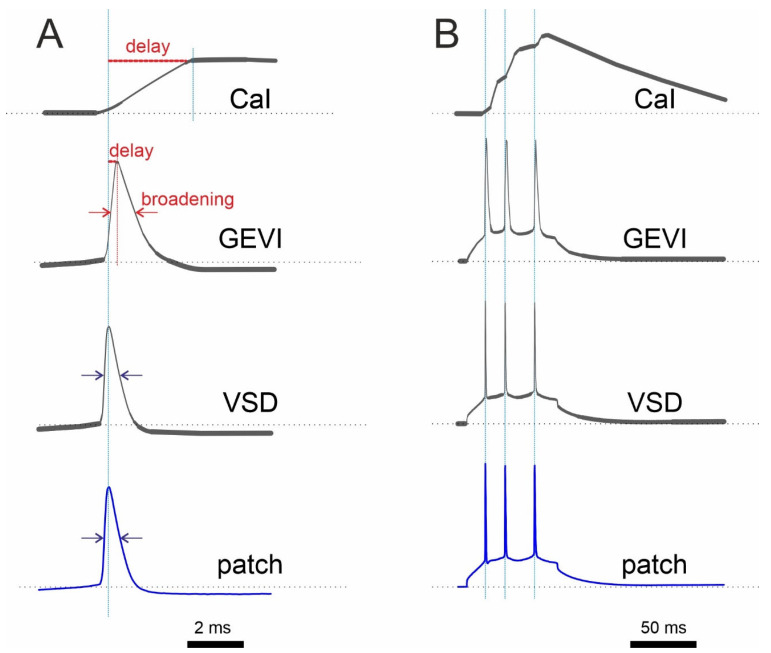
Schematic comparison of optical recording with a Ca^2+^ indicator (CaI), GEVI, and VSD with electrophysiological recording (patch) under ideal, low-noise conditions. (**A**) Single AP recordings show a relatively longer delay of the CaI signal compared to that of the GEVI signal. (**B**) In contrast to CaI, both GEVI and VSD show almost perfect discrimination of single APs in spike train. It is worth noting that the response of many GEVIs has more than one time constant. As a result, the amplitude of fast signals will not be reproduced in a linear fashion. Synthetic electrochromic VSDs, on the other hand, are linear without delay.

Classic fluorescent VSDs (JPW 1141 and analogues) are known to display the shapes of action potentials that are nearly identical to those that have been electrophysiologically recorded, with <1% signal broadening [43]. Detailed studies of the ANNINE-6 VSD have shown it to have nanosecond speeds [44]. New GEVIs have been claimed to be as fast as VSDs are, but they exhibit slight spike broadening (Figure 2). We measured the spike broadening of modern GEVIs (ASAP3, JEDI-2P, QuasAr1, QuasAr2, paQuasAr3, VARNAM, one SomArchon) from published data obtained on the pyramidal cells of cortical brain slices [30,45,46,47,48,49], as the cells from slices are known to have faster dynamics of action potentials than cultured neurons do [50]. The broadening measurements revealed a broadening range of 19–195%, the percentage added to the halfwidth of individual action potentials (i.e., the width of the action potential at half amplitude). Furthermore, the electrically recorded action potentials of these neurons [30,45,46,47,48,49] showed halfwidths of 1.9–9.4 ms, which are about two times slower than the normal halfwidth of pyramidal neurons of 0.5–1 ms, suggesting the interference of overexpressed GEVIs with the electrical properties of neurons.

In the last 5 years, GEVI works have been published in the highest-ranked journals including *Cell* [30,45,51], *Nature* [49], *Nature Methods* [52], and *Science* [53,54]. However, a meta-analysis of the literature indexed in PubMed revealed a more than twofold prevalence of studies using VSDs in comparison to those on GEVIs (Table 1). Thus, GEVIs have been used primarily by laboratories developing these new methods, which are not yet available for routine use by the majority of neuroscientists. Most of the neuroscience studies using VSDs have focused on population response recordings.

## 2. Invertebrate Studies with VSDs

### 2.1. Molluscan Examples of Successful VSD Employment

Mollusks can be used as conventional models of cellular mechanisms of learning and memory with a reduced number of neurons in the simpler nervous systems compared to those in mammalian brains. However, they show all the basic types of learning, including, but not limited to, Pavlovian conditioning, associative learning, sensitization, and fear conditioning. Thus, most of the work on mollusks concerned memory research.

There are two essential classes of VSDs used in mollusks for the detection of electrical activity: absorption VSDs (RH155 and JPW1131) and fluorescent VSDs (JPW1114 and di-4ANEPPS). Absorption dyes require an optical setup configuration that includes a photodiode array attached to a microscope in transmitted light mode. In contrast, fluorescent VSDs require a microscope in epifluorescent mode and a sensitive CCD camera. Both methods of optical recording need an electrically stabilized source of illumination for the reduction of background noise.

#### 2.1.1. Optical Recording of Multiunit Activity Evoked via Sensory Stimulation of Molluscan Ganglia

The optical recording of multi-unit activity with absorption VSDs (RH155 and JPW 1131) in transmitted light allowed the identification of large groups of individual cells that responded to physiologically relevant sensory stimulation during semi-intact preparation. With this technique, it is possible to measure the spiking activity of 30–50% of giant neurons (soma size up to 1 mm) in the ganglion of interest [55]. In total, up to 100+ neurons were simultaneously recorded within a single field of view [56]. The precise timing of individual spikes provided information about the specific response of classes of sensory neurons and their role in the behavioral reflex. Optical imaging revealed that ~90% of neurons that were activated during the gill retraction reflex were also activated during other types of movement [57].

However, the lack of information on connectivity and transmitters released by each neuron in the recorded pool prevented the precise reconstruction of the mechanistic picture of observed events. On the other hand, the data obtained were very helpful for statistical analysis. Overall, optical imaging data appear to be equivalent to the discriminated spike timings obtained via multiunit extracellular electrophysiological recording, but with more precise localization of the recorded neurons.

Optical data have shown that proprioreceptors do not contribute to the activity of withdrawal command neurons [58]. In addition, there was only a ~5% contribution from direct connections between sensory and motor neurons to the gill withdrawal reflex [59]. The responding neurons presumably included higher-order sensory neurons, tonically responding neurons, and a group of abdominal neurons whose activity correlated with the withdrawal position and speed [60]. The authors of a recent study on *Aplysia* [61] combined the classical RH155 transmitted light dye method with a standard CMOS camera, thus demonstrating that there is no need for an expensive, custom-made photodiode array. In addition to previous studies, new findings outlined different functional groups of motoneurons underlying operant conditioning [61].

**Figure 3:**
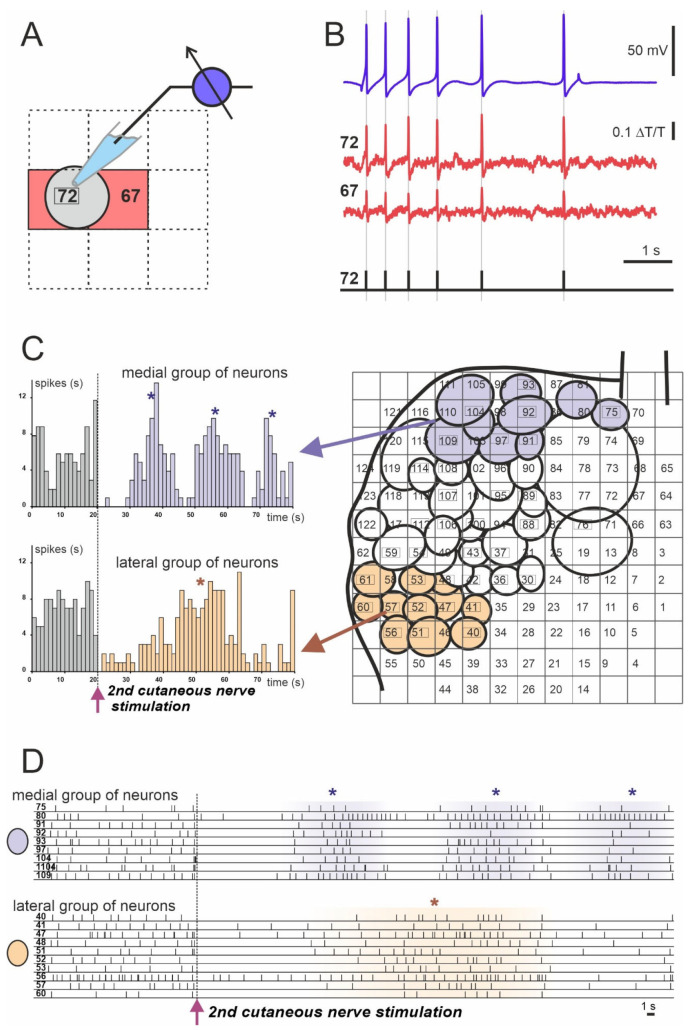
Example of spiking activity of molluscan neurons recorded using the absorption VSD (Rh155) and photodiode array. (**A**,**B**) Discrimination of recorded optically individual neuronal action potentials (APs). (**A**) Scheme of the cell’s body projection (gray circle) on the two photodiodes: #72 (cell number) and #67 (red squares). (**B**) Matching of single neuronal electrophysiological recording of the neuron #72 with a sharp electrode (blue trace) to the optical signals from photodiodes #72 and #67 (cell numbers are indicated by red squares). Individual AP timings are represented by vertical gray lines and black bars in the lower diagram. (**C**) Example of analysis of the population response of the *Helix* pedal serotonergic cells to electrical stimulation of the 2nd cutaneous sensory nerve. Projections of the cell bodies onto the photodiode array (right) with medial group (violet circles) and lateral group (light orange circles) are shown. Arrows point to the population response histograms (left) of individual groups. (**D**) Individual spiking of neurons used for the analysis and grouping shown in **C**. Each numbered line of spikes corresponds to one of the neurons drawn on the array in **C** (right). (*) Peaks of network firing. Data obtained by the authors.

Apart from the work on *Aplysia*, the use of absorption VSDs in similar experiments on *Helix* pedal ganglia allowed the visualization of the network response of giant serotonergic modulatory neurons to simulation of skin nerves [62,63]. Most neurons responded physically and tonically, while some neurons, such as the largest identified Pd2 cell, did not respond at all [62]. In addition, regularly oscillating clusters were identified among the tonically responding neurons (Figure 3).

Simultaneous recordings of the spiking activity of many neurons have been performed in several other species of gastropods, such as *Tritonia* and *Berghia* [64]. Recordings with absorption VSDs in the mollusk *Tritonia* revealed a number of neurons within the network that are involved in memory storage [65]. This study shifted invertebrate memory research from synaptic plasticity to neuronal assemblies and specific neurons that connect networks underlying specific behaviors [66].

#### 2.1.2. Odor-Evoked Modulation of Spontaneous Oscillations in the Procerebrum, an Olfactory Brain of Terrestrial Snails and Slugs

In contrast to other giant polyploid molluscan neurons, procerebral cells are very small, with a soma size of 5–7 µm; thus, they are comparable to the telencephalic neurons of mammals. Numerous procerebral neurons (up to 8 × 10^4^) are densely localized in compact cortex-like lobes of the procerebrum. The optical imaging with VSDs of the procerebral lobe composed of small neurons provided field potential recordings of the averaged activity of hundreds of neurons projected onto a single photosensitive pixel. Slow, spontaneously propagating oscillatory activity of the procerebrum was recorded using both the fluorescent VSD di-4ANEPPS [67,68] and the absorption VSD RH155 [69,70,71]. Odor presentation elicited various desynchronizations and modulations of the propagation velocity. Although these experiments did not demonstrate response specificity to the chemical structure of odorants presented, the negative feedback effect of oscillations on the withdrawal reflex was demonstrated via correlating the oscillatory activity with intracellular electrophysiological recordings from identified motoneurons [70].

#### 2.1.3. Visualization of Initiation and Propagation of AP in Subcellular Compartments of Giant Molluscan Neurons

The application of fluorescent VSDs to single giant neurons allowed the activity of an optically isolated neuron to be monitored, while the surrounding cells remained unstained. JPW1114 VSD provided higher sensitivity (up to ×50) than the absorption dyes did, resulting in an acceptable signal-to-noise ratio when it was recorded from the cell body and distant axonal branches [72].

Optophysiological experiments revealed several trigger zones of AP initiation on the axonal branches of identified mesocerebral giant neurons of *Helix* via tracing the AP propagation with VSD JPW1114 [73]. The activation of different trigger zones produced different propagation patterns [74]. Surprisingly, the propagating APs could reverse their direction at certain branching points [74]. In addition, VSD JPW1114 revealed the attenuation of APs at tiny axonal branches of the cerebral giant cells of freshwater snail *Lymnaea* that were inaccessible for microelectrode recording [75]. Moreover, voltage imaging demonstrated a relatively higher attenuation of AP amplitudes when APs were triggered at lower background potentials compared to that of those triggered at more depolarized membrane states [76].

### 2.2. Insect Examples of Successful VSD Employment

Most studies on insects have been conducted using the model of the fruit fly, *Drosophila melanogaster*. Some research has been conducted using the heterologous expression of *Drosophila* genes in mammalian cell cultures, such as screening for potential neurohormones [77]. Voltage fluctuations and oscillations in the antennal lobe in response to odor presentation or olfactory nerve stimulation have been demonstrated using optical imaging with VSDs in several insect models, including honeybees [78,79], bumblebees [80], hawk moths [81], and American cockroaches [82]. Signal processing in the sensory system has also been further investigated using voltage imaging in cockroach preparations [83].

The antennal lobe macroglomerular complex of the silkmoth, *Bombyx mori*, was optically imaged using a VSD under different conditions of nerve stimulation and environmental calcium concentrations, revealing the modular organization of the structure [84]. The serotoninergic modulation of antennal lobe responses to nerve stimulation was also demonstrated via optical imaging using VSD RH414 [85].

By staining the whole ganglia from crickets, crabs, and earthworms with VSD RH795, researchers were able to record the neural activity of an entire network with single-cell resolution [86].

### 2.3. Other Invertebrates

In a crab’s stomatogastric nervous system, two VSDs were validated for the simultaneous optical imaging of many neurons in the ganglion [87]. Di-4-ANEPPS showed a higher signal quality, but RH795 was found to be more suitable for long-term experiments due to its relatively low levels of photobleaching and phototoxicity. Furthermore, the combinatorial encoding of sensory information was found during stomatogastric ganglion preparation [88]. Using whole-ganglion optical imaging with Di-4-ANEPPDHQ and presenting sensory stimuli of two modalities, the authors found an overlapping combination of active neurons and signs of their responses to stimuli of each modality. A study from this lab using the same VSD in optically isolated single sensory neurons showed that signal integration depends on the location of ectopic spike initiation [89].

Staining with a FRET-based (donor–acceptor pair: coumarin–oxonol) VSD allowed Kristan’s lab to reveal the activity of many neurons in the segmental ganglion of the leech *Hirudo medicinalis* with single-neuron resolution during fictive behavior [90]. The central pattern generators for crawling and swimming were simultaneously recorded, and the candidate neuron responsible for the behavioral choice was detected. The optical imaging of the segmental ganglion in semi-intact leech preparation during local bending behavior revealed the role of local and CNS-wide inhibition in adjusting the behavioral gain [91]. Later, the new generation of PeT VSD developed in Miller’s lab was successfully applied to this preparation [92]. In further studies, the recording setup was improved to record both from dorsal and ventral surfaces of the segmental ganglion, allowing the majority of neurons in the ganglion (250 out of ~400) to be monitored with PeT VSD [93,94]. In the related species, *Hirudo verbana*, the same PeT dye showed differences between interneuron involvement in response to natural tactile stimuli and responses to electrical sensory neuron stimulation [95,96]. Recent work in the same model system has compared the functional network of the DE-3 motor neuron (recorded via optical imaging) during three different types of behavior during the ultramicroscopic imaging of synaptic network of this cell [97].

## 3. Vertebrate Studies with VSDs

### 3.1. Experimental Studies with VSDs (Mammals)

The voltage imaging of population responses can be used to monitor network development and signal propagation in preparations ex vivo [98,99] and in vivo [100,101,102]. Conventionally, optical mapping with VSDs is used in sensory physiology in various in vivo models [103,104,105,106,107,108].

In single-neuron configuration, VSD imaging has been successfully implemented to study the physiology of enteric neurons in ex vivo preparations [109,110,111]. In rodent brain slices, the propagation of dendritic potentials in pyramidal neurons was studied. This revealed how local synaptic inputs can “prepare” the cell for synchronous network activity [112,113].

#### 3.1.1. Imaging of Subcellular Compartments in Single Neurons

Typically, experiments in single mammalian neurons involve the loading of a single cell in acute rat or mouse brain slices with a VSD using a patch pipette (JPW1114 and its analogues). After loading and incubation at room temperature, the cell is re-patched with a dye-free patch pipette to record the cell and evoke action potentials either at room temperature or at near-physiological temperatures of 30–35 °C. This experimental arrangement allows electrophysiological traces to be matched with the optical traces of evoked signals averaged within the regions of individual dendrites or axonal branches. Overall, a complete picture of action potential propagation along the dendritic tree of principal pyramidal neurons was obtained [114,115,116,117,118,119,120]. Two-photon excitation even allowed action potential imaging to be performed with the dye di-2-AN(F)EPPTEA at higher optical resolution, which is good enough to resolve individual dendritic spines [121].

#### 3.1.2. Imaging of Action Potential Shape and Propagation in the Axons of Pyramidal Neurons

Most classical axonal imaging experiments are performed using the RedShirtImaging camera (12 × 80 binning) at 10 K frames per second with spatial resolution of 2–3 µm per pixel (60× objective and conventional epifluorescence microscope) [43,122]. To our knowledge, the highest temporal resolution of the optical imaging of the axon with a VSD was 20 K samples per second (confocal linescan at 20 K lines/s) [123]. However, since the axon is not straight, the line crossed the axon only in some areas (50–70% of the axon), without providing a complete picture of action potential propagation. The spike-triggered averaging of 5–10 evoked action potentials allowed them to slightly reduce the noise without strongly affecting the shape of the optical signal [123].

**Figure 4:**
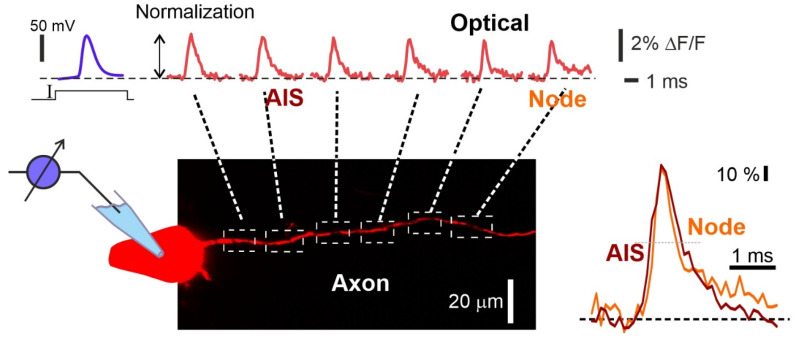
Visualization of action potential propagation in the axon of L5 pyramidal neuron stained with VSD JPW1114. Electrophysiological recording of the AP (upper left blue trace) and the corresponding spatial sequence of action potential signals (top red traces) recorded at axonal sites indicated by dashed white squares in the drawing below. Frames of the AP initiation and propagation. Normalized fine-scale signals from the axonal initial segment (AIS) and the first Ranvier node are shown (right bottom image). Optical recording obtained by the authors using a 63× lens (1 N.A.) and the RedShirtImging SMQ camera at 10 K fps. Data obtained by the authors.

In a general case, the axonal thickness (diameter: ~1 um) was smaller than the spatial resolution of the system. Furthermore, the quality of the optical trace is inferior than that of the electrophysiologically acquired recordings and did not allow the researchers to continue with the propagation of the action potential (AP) from pixel to pixel. Nevertheless, local spatial averaging of ~10–20 um regions along the axon allows the localization of the putative origin of AP initiation via analyzing the time-to-half-amplitude of optically recorded APs [43]. Although the shapes of the optically recorded APs were different, their depolarization phases were thought to reflect AP propagation driven by the activation of fast voltage-gated sodium channels (Figure 4). In addition, this type of optical recording allows to reveal the origin of the AP and to follow its propagation along the axon [43].

In addition to the propagation analysis of depolarizing phases of the AP, the optically recorded AP shape provides information about the AP duration (width), which is largely contributed by voltage-gated potassium channels. The imaging of pyramidal neurons with VSDs revealed that distal parts of the axon and its proximal collaterals have a higher density of fast A-type potassium channels compared to that of the soma, as the AP showed a strong broadening upon the application of specific blockers [124]. In addition, voltage imaging allowed the visualization of saltatory conduction patterns in the myelinated axons of pyramidal neurons, as well as the monitoring of AP characteristics, which were imaged as dynamic shapes of the transmembrane potential [122]. The voltage imaging of AP shapes in the axon of Purkinje cells revealed a decay of some spikelets as they moved forward along the axon at firing frequencies higher than ~250 Hz [125], while the APs originated from the distal part of the axonal initial segment [126]. Single Purkinje neurons can be filled with synthetic VSDs, and then used for in vivo voltage imaging to detect EPSPs, as well as dendritic spikes [35,36].

#### 3.1.3. Imaging of Field Potentials in Acute Slices

In addition to single cell resolution, when many cells are stained with a VSD, voltage imaging allows the field potentials of neuronal networks to be recorded using a lower magnification. Both photodiode array and NeuroCCD (or similar) cameras give very similar results for brain slices treated with water soluble dyes (i.e., RH155/RH482, [127,128]). Typically, neurons are stained from the outside by adding water-soluble dissolved dyes to the bath or perfusion system. Hydrophobic dyes, such as di-12-ANEPPQ and di-8-ANEPPS, can be used for staining via the biolistic delivery of dye-coated microbeads with a gene gun [14,129,130]. Imaging with VSDs was used to study pharmacologically induced wave propagation in acute brain slices of the hippocampus [128,131] and neocortex [127], which are considered to be seizure-like oscillations.

#### 3.1.4. Imaging in Studies of Neurological Disorders

Currently, VSDs are considered as promising candidates for the imaging and localization of epileptic foci as a targeting tool for surgical excising as a part of the treatment of drug-resistant epilepsies [132]. Recently, Kang et al. [133] used the near-infrared cyanine VSD, IR780 perchlorate, for the transcranial visualization of seizures locally induced by chemoconvulsant injection in a rodent model in vivo. In addition, the optical recording of large populations of neurons with VSDs is quite efficient for studying seizures and screening of antiepileptic drugs [134]. For example, evoked responses in acute brain slices have been used to characterize the pharmacological modulation of neuronal activity induced by antiepileptic drugs [135,136].

#### 3.1.5. Other Applications of Multiunit Optical Recording

Similar to invertebrate works, the multi-unit recording of single-neuronal activity was used to describe individual responses of interconnected ganglia neurons in the guinea pigs’ enteric nervous systems to agonists and antagonists of nicotinic receptors [137,138]. In combination with immunohistochemical labeling, these data allowed the mapping and identification of pharmacological profiles of individual neurons.

### 3.2. Lower Vertebrate Studies with VSD

Voltage-sensitive dyes have been used to record electrical activity in the brain of various lower vertebrates. In the zebrafish *Danio rerio* model, Di-4-ANEPPS was used to stain the cerebellum and spinal cord in juvenile preparations during electrical and pharmacological stimulation [139,140]. The excitatory and inhibitory population responses were observed at the single cell resolution. In another zebrafish model, signal processing in the olfactory bulb was investigated [141]. The axons of olfactory receptor neurons were labeled anterogradely with Di8-ANEPPQ, allowing the optical imaging of afferent axons and across the array of glomeruli during presentations of different odorants.

In an in vitro preparation of brains of filefish Stephanoplepis cirrhifer, the single cell resolution of optical imaging was achieved in the thalamic nucleus with the Di-4-ANEPPS dye [142]. In the retina of *Carassius auratus* (goldfish), FluoVolt VSD imaging allows the recording of single horizontal cells and the detection of spontaneous calcium spikes in intact neurons [143]. Population responses of the optical tectum have also been recorded in vitro [144]. In an in vivo model of juvenile rainbow trout, *Oncorhynchus mykiss*, population responses of the optic tectum to light stimuli were recorded, and retinal projections were described using the RH-414 VSD [145,146]. In mormyrid fish, *Gnathonemus petersii*, electrosensory lobe neurons have been studied with VSD di-4-ANEPPS, revealing properties of AP generation and dendritic backpropagation similar to those of mammalian neurons [147].

Early studies using optical imaging with absorbance VSDs used two different frog preparations, *Rana sp*., sympathetic ganglia [148] and olfactory bulbs, where population responses to odors were successfully optically recorded [149]. In vitro spinal cord and brainstem preparations of the same genus of frog were used to study respiratory rhythm via optical imaging with the fluorescent dye, di-2-ANEPEQ [150]. In the in vitro preparation of the sacculus of the same genus of frog, subthreshold potential oscillations in the membrane of hair cells that cause electrical resonance were detected using the VSD di-3-ANEPPDHQ [151]. In the frogs’, *Rana sp*., neuromuscular junction, the width and shape of the action potentials in the nerve terminal were measured via optical imaging with BeRST 1 VSD [152]. It was possible to distinguish three different regions in the nerve terminal by changes in the AP shape, explaining the proximal–distal gradient in acetylcholine release observed in electrophysiological experiments.

In another amphibian species, the salamander, *Ambystoma tigrinum*, the optical recording of the olfactory bulbs’ surface during odor presentation was performed, and the spatiotemporal propagation of the electrical signal was characterized [153].

In a semi-intact CNS preparation of pond turtles, *Pseudemys scripta*, the use of absorption VSD for recording the single-spike activity of pyramidal neurons was validated via simultaneous optical and electrophysiological recording [154]. Further work using the same merocyanine absorption dyes and the fluorescent styryl dye RH795 in the turtle visual system explored the potential for recording population responses [155,156,157]. In this model preparation, the first evidence for the modal operation of cortical networks was found [158].

Another semi-intact reptile brain model used in optical imaging is the olfactory system of turtles, *Terrapene carolensis*. Turtles were chosen for the imaging of odor-evoked responses of intact olfactory bulbs in semi-intact preparations because they are similar to mammals, but allow experiments to be conducted at room temperature [159,160]. In the olfactory bulbs of these turtles, voltage oscillations have been found to spread across the surface in response to odorant stimulation [159,160]. Optical recording with the fluorescent VSD RH414 combined with electrophysiological field potential recording allowed the researchers to distinguish three different oscillation frequencies evoked by odor presentation in the olfactory bulb. This preparation allows the study of bulbs responses to repetitive stimuli [161] and during olfactory Pavlovian conditioning with two different odors that presented ipsi- and contralaterally [162].

The third model used in the turtles’ brain for optical imaging is the intact cerebellum in vitro. This is convenient because the cerebellum in reptiles is thin and unfoliated, and its entire surface can be stained with VSD [163,164,165]. The vestibular stimulation in this model can be mimicked via the electric stimulation of VII cranial nerve or the inferior olive.

In a few studies, VSD has been applied to avian CNS as a model object to study mechanisms of brain development [166] and visual processing in the Wulst (analog of mammalian striate cortex) [167] and the brainstem [168,169].

## 4. Experimental Studies with GEVIs in Vertebrates

The voltage-sensitive fluorescent protein was first developed over 20 years ago and exploited the voltage-dependent conformational changes around the fourth transmembrane segment (S4) of voltage-gated K+ channel (Kv) proteins and used either FRET [170] or a permuted fluorescent protein [171]. It did not show satisfactory response characteristics when it was expressed in mammalian cells due to poor targeting to the plasma membrane [172]. The next generation of GEVIs based on the voltage-sensing domain (VSD) of *Ciona intestinalis* voltage sensor-containing phosphatase (Ci-VSP) [19] (VSFP2.1, [173] VSFP2.3 and VSFP3.1, and [174] VSFP2.42) made it possible to obtain a reliable optical readout of the voltage signals from neurons in brain slices and in living mice [175,176,177]. Initial studies have demonstrated the ability of GEVIs to respond to voltage fluctuations by displaying optical signals, but the dynamic characteristics and signal amplitudes were modest. Therefore, the development of the latest GEVIs has been further optimized to meet the physiological goals [47,49,178].

### 4.1. Lower Vertebrate Studies with GEVIs

The only genetically traceable organism between invertebrates and mammals is the zebrafish, *Danio rerio*. The authors of a few studies have used this model for the optical imaging of GEVIs. The first attempts to express GEVIs in zebrafish were only partially successful [179]. Although later, the protein was successfully expressed in neurons and targeted to the cell membrane, ASAP1 was reportedly not expressed in neurons and the fluorescence of Bongwoori was prone to spontaneous fluctuations. Recently, ASAP1 was successfully expressed in the cerebellum and spinal cord of zebrafish larvae in vivo, allowing population and limited single neuron voltage recording [139].

The most successful GEVI study in zebrafish to date used a chemigenetic approach to increase the brightness of the Ace-based protein. Different variations of Voltron based on Ace and bright external synthetic fluorophore instead of fluorescent protein were used to successfully record single spikes and subthreshold voltage perturbations in larval zebrafish in vivo [180]. Optical imaging with Voltron in the tegmental area of the midbrain simultaneously recorded up to 11 neurons during a light-induced swimming ‘bout’. The electrical activity of these neurons varied in latency and sign of response before tail movement, revealing three classes of neurons that comprise the network.

### 4.2. Experimental Studies with GEVIs in Mammals

Genetically encoded voltage indicators (GEVIs) have revolutionized the field of neuroscience by offering non-invasive and high-sensitivity tools for studying neural activity in real-time. GEVIs enable the study of individual neurons and neural circuits in a controlled environment, providing researchers with insights into subthreshold synaptic activity, action potentials, and spatiotemporal patterns of activity that are difficult to observe with traditional methods. Major advances in the use of GEVIs have been made in mammals due to the availability of targeted gene expression methods that have been best developed for mammalian organisms.

#### 4.2.1. Experimental Works with GEVIs in Acute Brain Slices

Recent studies have demonstrated the potential of GEVIs in acute brain slices. For instance, Li et al. [181] used ASAP3, a GEVI based on the voltage-sensing domain of the phosphatase Ci-VSP from *Ciona intestinalis*, to monitor electrical activity from multiple neurons in brain slices. They observed the reliable detection of subthreshold synaptic activity and action potentials, as well as the propagation of activity across neuronal populations. Similarly, VSFP2.1, VSFP2.3, VSFP3.1, and VSFP2.42 have been used to obtain reliable optical readouts of voltage signals from neurons in brain slices and living mice [19,173,174]. These GEVIs have been applied to study neural oscillations and seizures in the hippocampus [175].

Moreover, GEVIs have also been used to study neural waves, perform single-cell recordings, and reveal lateral inhibition in acute brain slices. Chiang et al. [182] used VSFP-Butterfly 1.2 to study fast and slow neural waves during seizures in the hippocampus. Similarly, Quicke et al. [38] employed the same indicator for single-cell recordings in cortical layers 2/3 of the pyramidal neurons, while Nakajima et al. [183] used ArcLight to reveal lateral inhibition in hippocampal slices. These studies demonstrate the enormous potential of GEVIs in acute brain slices, and their increasing use is likely to accelerate the pace of discovery in neuroscience research.

#### 4.2.2. Experimental Works with GEVIs In Vivo

Several studies demonstrated the capabilities of VSFP Butterfly 1.2 in recording cortex-wide electrical activity in vivo with a high spatiotemporal resolution [184,185,186,187]. Quicke et al. [188] combined light field microscopy with Butterfly 1.2 to enable the imaging of somatic and dendritic structures.

Chi-VSFP was used on transgenic mice to reveal novel cortical signatures of awake somatosensory processing, including cortex-wide hyperpolarizing activity following the initial sensory-evoked depolarizing response [189]. ASAP3 enabled single-cell voltage imaging in vivo preparations, as demonstrated in rodent studies [45,190]. The development of JEDI-2P improved the performance of the ASAP family of indicators, allowing for the recording of voltage dynamics of individual cortical neurons in awake mice for more than 30 min [30].

PostASAP, a modified version of ASAP1 with enhanced sensitivity and enriched expression in the spines, allowed the measuring of membrane potentials in spines and dendrites from pyramidal neurons in the somatosensory cortex of mice during spontaneous activity and sensory stimulation [54]. By using two-photon optogenetics combined with voltage imaging, the authors found that individual spines can be activated independently, indicating that dendritic spines are also elementary electrical compartments for synapses.

ArcLight, another GEVI, was first reported for in vivo use in 2015 by Storace et al. [191]. Injections of an AAV1 vector resulted in widespread labeling of mitral and tufted neurons in the olfactory bulbs of mice. Later studies in the barrel cortex [192], the olfactory system [193], and on thalamocortical signaling [194] confirmed the feasibility of the sensor for in vivo voltage imaging. Studies in the olfactory system showed that ArcLight had significantly faster temporal kinetics than genetically encoded calcium reporters did [191,195], but a lower signal-to-noise ratio. Platisa et al. [37] reported the first transgenic mouse lines with ArcLight expression restricted to either olfactory receptor neurons or a subpopulation of interneurons located in the granule and glomerular layers of the olfactory bulb, allowing the first in vivo measurements of membrane potential changes in these neurons. Recently, Bando et al. [196] developed a new ArcLight-based sensor, ArcLight-ST, for better measuring subthreshold membrane potentials with a cellular resolution in vivo.

Several studies demonstrated the feasibility of microbial rhodopsin-based GEVIs as a tool for single cell recording in vivo [47,49,178]. Improving QuasAr2 led to a new construction, paQuasAr3-s [47] with enhanced SNR that enabled single-neuron optical voltage measurements in CA1 region of the hippocampus. Imaging from awake, head-fixed mice walking on a motorized treadmill revealed that forced walking decreased the mean spike rate of neurons in *stratum pyramidale* but increased the of *stratum oriens* [47]. SomArchon [49] was first developed to be compatible with blue light-driven optogenetics for single-cell recording in vivo. Later, optogenetic techniques for resolving excitatory contributions from inhibitory contributions to subthreshold voltage combined with voltage imaging with SomArchon revealed lateral inhibition within L1 in the barrel cortex [51]. Another important study demonstrated the possibility of genetically targeting GEVIs with different fluorescence spectra and optical spike polarities in distinct neuron types [53]. The authors previously introduced green and red fluorescence resonance energy transfer (FRET)-opsin GEVIs Ace-mNeon and VARNAM that exhibit fluorescence decreases during action potentials [48]. Further, the sensors were improved (Ace-mNeon2 and VARNAM2) and were shown to have an increased voltage sensitivity. Along with newly identified the respective reverse response-polarity variants, pAce and pAceR, these sensors allowed the dual-polarity and dual-color real-time voltage imaging from up to three neuron types. This technique provides the possibility of studying the contribution of distinct cell classes to various electric processes in the brain, such as local field potentials in the hippocampus [53].

#### 4.2.3. GEVIs in Studies of Neurological Disorders

The main advantage of the voltage-regulated imaging of neuronal populations with GEVI is the ability to target the indicator to specific groups of cells. This can be achieved using either pan-neuronal promoters or group-specific promoters that restrict indicator expression to a specific group of cells, e.g., all neurons or all parvalbumin-specific interneurons [197]. On the other hand, the loading of cells with a GEVI requires genetic interference for the expression of a foreign protein; therefore, experiments on pure wild-type cells are not possible. In contrast to VSDs, GEVIs have not been considered for imaging seizure manifestations in human epileptic patients [132]. However, GEVI-expressing neuronal populations in rodents provide very useful in vivo and ex vivo models for studying epileptic-like activity. The voltage-sensitive fluorescent protein Butterfly 1.2 was used to image slow propagating voltage waves in an in vitro rodent model of the epileptic hippocampus in combination with simultaneous Ca^2+^ imaging using a bath-applied Ca^2+^-sensitive dye [182]. NMDA-independent slow waves of both Ca^2+^ and voltage spikes propagated in ephaptic-like manner, which was similar to human epileptiform activity [182]. In addition, GEVI ArcLight-MT was used to map the propagation of epileptic seizures in a mouse model in vivo using two-photon voltage imaging, allowing seizure progression and local voltage dynamics to be revealed [196]. Recently, Zhu et al. [198] studied synaptic dysfunction in Alzheimer’s disease using population voltage imaging on the slices of chi-VSFP transgenic animals. In this study voltage imaging revealed early-onset hyperexcitability in L2/3 pyramidal neurons, with significantly longer halfwidths of voltage transient in the Alzheimer’s disease group compared to those of the controls and older age groups.

#### 4.2.4. Further Perspectives on the Use of GEVIs in Mammalian Systems

Nowadays, GEVIs have become an important tool for monitoring neuronal activity, particularly due to their high spatial and temporal resolutions, which make them an excellent choice for studying the dynamics of large neural networks. In addition, a growing number of in vivo studies in recent years strongly suggest that potentiometric imaging technology demonstrates a clear trend toward in vivo experimentation in brain research, driven by the desire to combine it with genetic engineering techniques (Table 2). However, older and relatively slower probes of the Arlight and Butterfly 1.2 families have been most commonly used in, in vivo studies focused primarily on basic neuroscience research (Table 2). As evidenced by their popularity in experimental studies, Red Shirt Imaging NeuroCCD and Hamamatsu ORCA-Flash 4.0 cameras have been most often used for voltage imaging with GEVIs, which are also well suited and commonly used with VSDs.

GEVI studies are thought to provide researchers with readily available animal models for in vivo potentiometric experiments, eliminating the need for staining with VSDs or other dyes. Thus, the introduction of GEVIs into routine neuroscience research is often expected to result in genetically engineered animals with a potentiometric probe in the brain, which is ready for use either for in vivo imaging or as a source of acute brain slices or primary neuronal cultures.

## 5. Invertebrate Works with GEVIs

The first successful demonstration of GEVI expression in the intact neural system of an invertebrate was made in a fruit fly, *Drosophila melanogaster* [202]. In this pioneering study, using ArcLight, the single spikes and subthreshold potentials were recorded from cell bodies and neurites in vivo. The authors demonstrated the power of the method via recording spontaneous rhythmic activity of circadian clock neurons and odor-evoked activity of the olfactory neurons.

By pan-neuronally expressing ArcLight in the fruit fly nervous system and optically recording nearly the whole brain, the researchers achieved the resolution of neuropil structures and were able to extract spike-like activity from subneuropil compartments in response to light and odor in vivo [203]. The large-scale method developed is analogous to whole-brain fMRI recordings in humans, but with the advantage that the fly can walk during the recording. The authors also used serotonergic and dopaminergic neuron promoters to express ArcLight only in specific neuron types.

In the *Drosophila* neural system, ASAP-based sensors have been used to record electrical events at the cellular and subcellular levels during visual stimulation in vivo [204,205]. In specific visual neurons, responses to ON and OFF light stimuli were recorded in the soma and neuropil synaptic buttons, revealing pre- and postsynaptic voltage dynamics. In combination with calcium imaging, this allows the localization of contrast selectivity coding in the neural network under study. Further work from this lab in the fruit fly visual system has been conducted with the new improved GEVI JEDI-2P, which provides a 60% larger response to two-photon excitation and improved photostability [30]. Using ASAP2f, the mechanism of directional selectivity of visual stimuli via linear input summation in the fruit flies was shown [206].

The voxel timing analysis method was proposed to extract the shape of fast repetitive electrical events from the signal via slow frame rate imaging and was successfully tested on dendritic arbors of *Drosophila* Mi1 visual neurons, demonstrating the potential of the ArcLight sensor [207]. Ace-based chemigenetic GEVI Voltron was suitable for recording single-spike events in *Drosophila* dopamine neuron dendrites [180].

The neuromuscular junction in *Drosophila* was studied using Arch voltage imaging [208]. By comparing the optical signal with electrophysiology, the Arch sensor was shown to reliably resolve the shape and width of the AP without altering acetylcholine release in this model. Using a shaker mutant line of fruit flies and altering the extracellular calcium concentration, the authors demonstrated the role of BK channels and synaptic Ca^2+^ in AP repolarization.

Recently, a new panel of Ace-based GEVI was invented, and the authors showed that it allows recording of individual spikes in axonal regions of specific neuronal types in *Drosophila* during odor presentation in vivo [48,53,178]. Recently, a new promising method of two-photon recording from single neurons in freely behaving *Drosophila* larvae was invented, and the activity of sensory and motor neurons during behavior was demonstrated [209].

In the nematode, *Caenorhabditis elegans*, the in vivo membrane potential recording of a single genetically transfected AWC-ON olfactory neuron with Archer1 GEVI was achieved during odor presentation, albeit below the single spike resolution [210]. Recently, a series of microbial rhodopsin GEVIs were investigated in the model organism, *C. elegans* [211]. In this study, the QuasAr sensor was expressed in two RIM and cholinergic neurons, which also achieved the monitoring of electrical activity below the single spike resolution.

## 6. Conclusions Remarks

The optical imaging of neuronal activity with a potentiometric probe (VSD or GEVI) is technically difficult and limited by several methodological constraints that determine its applicability in a given type of experiment. Thus, the popularity of this method is far from being comparable to patch clamp or similar routine methods in neuroscience research. From the GEVI literature analysis, it is clear that the field is developing more in a methodological manner than a scientific one. More than twice as many studies have been conducted with VSDs than those with GEVI. As can be seen from the majority of the papers, most of them are either methodological or reviews. In addition, GEVI research on neurons comes mostly from the labs involved in GEVI design and development. However, over the past 50 years, potentiometric imaging has been given credit for being able to address key questions in neuroscience via simultaneously recording most or many neurons. Therefore, potentiometric imaging has the potential to provide unique information that cannot be obtained via other methods. With the increasing adoption of these technologies, one can anticipate gaining deeper insights into the complexities of neural circuitry and the mechanisms underlying brain function.

## Figures and Tables

**Table 1:** Meta-analysis of the recent literature on the field of voltage sensors.

A. PubMed Query: Voltage [All Fields] and “Sensitive” [All Fields] and “Dye” [All Fields]							
Year	Total	Methodical	Population Response	Single Neurons and Compartments	Review	Non-Neuroscience	Non-Relevant
2022	23	0	9	2	1	5	6
2021	44	8	11	3	0	16	6
2020	61	7	22	5	2	17	8
2019	58	6	25	2	4	11	10
2018	54	6	24	4	0	10	10
**B.** **PubMed query: (“genetically encoded voltage indicator”) OR GEVI**							
**year**	**total**	**methodical**	**population response**	**single neurons and compartments**	**review**	**non-neuroscience**	**non-relevant**
2022	21	6	4	2	0	4	5
2021	19	6	7	0	4	0	2
2020	20	6	0	2	2	5	5
2019	18	6	0	1	4	2	5
2018	16	5	2	2	1	2	4

**Table 2:** Overview of recent applications of GEVIs in neuroscience research in rodent models. Spatial resolution is divided into the following categories (from coarser to finer): field (field potential recording, individual cells are not resolved), cell, soma (partial recording of the neuron), dendrites (large cellular compartments), and spines (fine compartments of the dendrites of spiny neurons). Green highlighted references: primary focus on the basic neuroscience research. Yellow highlighted references: methodological focus with significant data obtained that are relevant to the field of basic neuroscience. The name of the potentiometric indicator, the recording method (instrumentation) and the frame rate (frames per second and temporal resolution of the recording) are shown.

Resolution	Model	Indicator	Recording	Frame Rate	Source	Year
Cell	culture, acute brain slices	Ace2N-mNeon	EMCCD	5000	[178]	2015
	In vivo		sCMOS	500–1000		
Soma	culture, in vivo	ASAP2, ASAP3	ORCA-Flash 4.0	100	[45]	2019
Cell	culture, acute brain slices, in vivo	QuasAr3, paQuasAr3)	ORCA-Flash 4.0	500–1000	[47]	2019
Dendrites, Spines	culture, in vivo	postASAP	NeuroCCD SM256	60	[54]	2022
Dendrites	culture	QuasAr2	ORCA-Flash 4.0	484–1058	[199]	2022
Field	acute brain slices	ArcLight	NeuroCCD SMQ	1000	[200]	2018
Field	acute brain slices	VSFP-Butterfly 1.2	ORCA-Flash 4.0	200	[182]	2018
Soma	acute brain slices	SomArchon	EMCCD iXON, sCMOS Zyla 4.2	1000	[49]	2019
	in vivo		ORCA-Flash 4.0	390–900		
Cell, Soma, Dendrites	acute brain slices	ASAP3	Two-photon microscope	2000–10,000	[181]	2020
Field, Cell, Denrites	acute brain slices, in vivo	ArcLight-ST, Kv-ArcLight-ST	ORCA-Flash 4.0	30–1000	[196]	2021
Field	acute brain slices	ArcLight	NeuroCCD SMQ	1000	[183]	2021
Field	acute brain slices	ArcLight, Bongwoori-R3, Bongwoori-Pos6	NeuroCCD SMQ	1000	[197]	2021
Field	acute brain slices	ArcLightD, chi-VSFP, Archon1	NeuroCCD SMQ	1000	[201]	2021
Field	acute brain slices	chi-VSFP	NeuroCCD SMQ	1000	[198]	2022
Field	in vivo	VSFP-Butterfly 1.2	CCD Sensicam	50	[186]	2014
Field	In vivo	ArcLight	NeuroCCD SM256	125	[191]	2015
Field	in vivo	VSFP-Butterfly 1.2	CCD Sensicam	50	[185]	2016
Field	in vivo	ArcLight	NeuroCCD SM256	25–125	[193]	2017
Field	in vivo	VSFP Butterfly 1.2	CCD Sensicam	50	[187]	2017
Field	in vivo	chiVSFP	CMOS Basler	150	[189]	2018
Field	in vivo	ArcLight	NeuroCCD SM256	40–125	[195]	2019
Field, Cell	in vivo	SomArchon	ORCA-Flash 4.0	1000	[51]	2020
Cell	in vivo	ArcLight	NeuroCCD SM256	50–250	[37]	2022
Field	In vivo	ArcLight	CCD MiCam2 HR	200	[194]	2022
Cell	in vivo	Ace-mNeon2, pAce, pAceR and VARNAM2	ORCA-Flash 4.0	50	[53]	2022
Cell	in vivo	JEDI-2P	Two-photon microscope	2525–3333	[30]	2022
